# A longitudinal review of national HIV policy and progress made in health facility implementation in Eastern Zimbabwe

**DOI:** 10.1186/s12961-018-0358-1

**Published:** 2018-09-21

**Authors:** Malebogo Tlhajoane, Tidings Masoka, Edith Mpandaguta, Rebecca Rhead, Kathryn Church, Alison Wringe, Noah Kadzura, Nimalan Arinaminpathy, Constance Nyamukapa, Nadine Schur, Owen Mugurungi, Morten Skovdal, Jeffrey W. Eaton, Simon Gregson

**Affiliations:** 1 Department for Infectious Disease Epidemiology, Imperial College London, Norfolk Place, London, W2 1PG United Kingdom; 2 Biomedical Research and Training Institute, Harare, Zimbabwe; 3 Department of Population Health, London School of Hygiene & Tropical Medicine, London, United Kingdom; 4 Ministry of Health and Child Care, Harare, Zimbabwe; 5 Department of Public Health, University of Copenhagen, Copenhagen, Denmark

**Keywords:** HIV, Health services, Health policy, Implementation, Zimbabwe, Antiretroviral therapy

## Abstract

**Background:**

In recent years, WHO has made major changes to its guidance on the provision of HIV care and treatment services. We conducted a longitudinal study from 2013 to 2015 to establish how these changes have been translated into national policy in Zimbabwe and to measure progress in implementation within local health facilities.

**Methods:**

National HIV programme policy guidelines published between 2003 and 2013 (*n* = 9) and 2014 and 2015 (*n* = 5) were reviewed to assess adoption of WHO recommendations on HIV testing services, prevention of mother-to-child transmission (PMTCT) of HIV, and provision of antiretroviral therapy (ART). Changes in local implementation of these policies over time were measured in two rounds of a survey conducted at 36 health facilities in Eastern Zimbabwe in 2013 and 2015.

**Results:**

High levels of adoption of WHO guidance into national policy were recorded, including adoption of new recommendations made in 2013–2015 to introduce PMTCT Option B+ and to increase the threshold for ART initiation from CD4 ≤ 350 cells/mm^3^ to ≤ 500 cells/mm^3^. New strategies to implement national HIV policies were introduced such as the decentralisation of ART services from hospitals to clinics and task-shifting of care from doctors to nurses. The proportions of health facilities offering free HIV testing and counselling, PMTCT (including Option B+) and ART services increased substantially from 2013 to 2015, despite reductions in numbers of health workers. Provision of provider-initiated HIV testing remained consistently high. At least one test-kit stock-out in the prior year was reported in most facilities (2013: 69%; 2015: 61%; *p* = 0.44). Stock-outs of first-line ART and prophylactic drugs for opportunistic infections remained low. Repeat testing for HIV-negative individuals within 3 months decreased (2013: 97%; 2015: 72%; *p* = 0.01). Laboratory testing remained low across both survey rounds, despite policy and operational guidelines to expand coverage of diagnostic services.

**Conclusions:**

Good progress has been made in implementing international guidance on HIV service delivery in Zimbabwe. Further novel implementation strategies may be needed to achieve the latest targets for universal ART eligibility.

## Background

The Joint United Nations Programme for HIV/AIDS (UNAIDS) estimates indicate that, in 2015, there were 36.7 million people living with HIV (PLHIV) worldwide, of whom 66% lived in sub-Saharan Africa [1]. Antiretroviral therapy (ART) has been shown to be highly efficacious in reducing mortality, improving the immunological status of PLHIV and reducing onward transmission of HIV [2–5]. As evidence of these benefits has grown, WHO recommendations have been revised over time to expand eligibility for ART. Initial guidance on the scale-up of ART in resource-limited settings suggested that treatment be initiated for all individuals with a CD4 count ≤ 200 cells/mm^3^ or at clinical stages III or IV [6]. In 2010 and 2013, the CD4 count threshold for ART initiation was revised upwards to ≤ 350 cells/mm^3^ and ≤ 500 cells/mm^3^, respectively [7, 8]. Most recently, in 2015, WHO released new guidelines recommending ART initiation for all PLHIV regardless of their CD4 cell count or clinical stage [9, 10]. To drive progress in implementing the latest WHO guidelines, UNAIDS set the 90–90–90 treatment targets, specifying that 90% of PLHIV should be diagnosed and aware of their sero-status, 90% of those diagnosed should be receiving ART and 90% of those on ART should be virologically suppressed by the year 2020 [11].

Generally, countries in sub-Saharan Africa (including Zimbabwe) and elsewhere have taken up these recommendations and adopted novel strategies to increase uptake of HIV testing and treatment services [12–14]. Key strategies have included expansion of prevention of mother-to-child transmission (PMTCT) programmes and the introduction of immediate and lifelong ART for all pregnant and breastfeeding women (Option B+). Other important health system innovations have included (1) increased use of community-based HIV testing, (2) decentralisation of treatment programmes from hospitals to clinics, and (3) task-shifting of HIV care from doctors to nurses [15–18]. These changes, supported by a global effort to mobilise resources, have succeeded in substantially increasing uptake of treatment services. By June 2016, approximately 50% of all PLHIV worldwide were receiving ART, up from 6.9% in 2005 [1]. However, despite these changes, many PLHIV continue to initiate ART at low CD4 cell counts, with advanced clinical symptoms, indicative of AIDS-defining illnesses [19, 20]. Previously, challenges in the delivery of HIV services have been attributed to constrained health system infrastructure, primarily in remote and rural areas. This included insufficient laboratory facilities, limited availability of human resources, and limitations in the availability of drugs and other medical supplies [18, 21–23]. A 2014 national evaluation of HIV policy implementation in Zimbabwe found health worker availability and competence, and the ART supply chain, to be additional difficulties [24]. All of these factors are likely to be compounded by the growth in patient numbers as a result of expanded ART eligibility.

In Zimbabwe, HIV prevalence declined in the early 2000s due to reductions in sexual risk behaviours, leading to a decrease in HIV incidence and AIDS-related mortality [25, 26]. HIV prevalence continued to fall following the introduction of the public sector ART programme – from 19.2% in 2004 to 14.7% in 2015 [26, 27]. Nevertheless, despite these successes and a major expansion of the HIV treatment programme in recent years, only 72% of adults eligible for ART were receiving treatment at the end of 2015 [24, 28, 29].

Health information systems data and surveys are being used to track progress towards meeting the UNAIDS targets. However, in-depth case studies are also needed to understand the factors facilitating and obstructing progress towards meeting these targets. In particular, studies are needed to document how high-level recommendations are translated into detailed policies and strategies, and to evaluate progress in their implementation at the local level. In this paper, we use data from policy reviews and health facility surveys to (1) evaluate changes in national HIV testing and treatment strategies between 2013 and 2015, and (2) to measure progress in implementing these policies at local health facilities in east Zimbabwe over the same time frame. Policies and strategies considered include those relating to HIV testing and counselling (HTC), PMTCT, and ART initiation, retention and patient monitoring.

## Methods

### Study components and research populations

This study is comprised of two components. We first perform a review of national policy documents on HIV care and treatment services in Zimbabwe in 2013 and updated in 2015. Secondly, we analyse data from two rounds of a health facility survey in two districts in Manicaland, Zimbabwe’s eastern province. The survey was conducted in 2013 and 2015 to measure changes in implementation of national HIV policies and strategies.

### Policy reviews

**Fig. 1:**
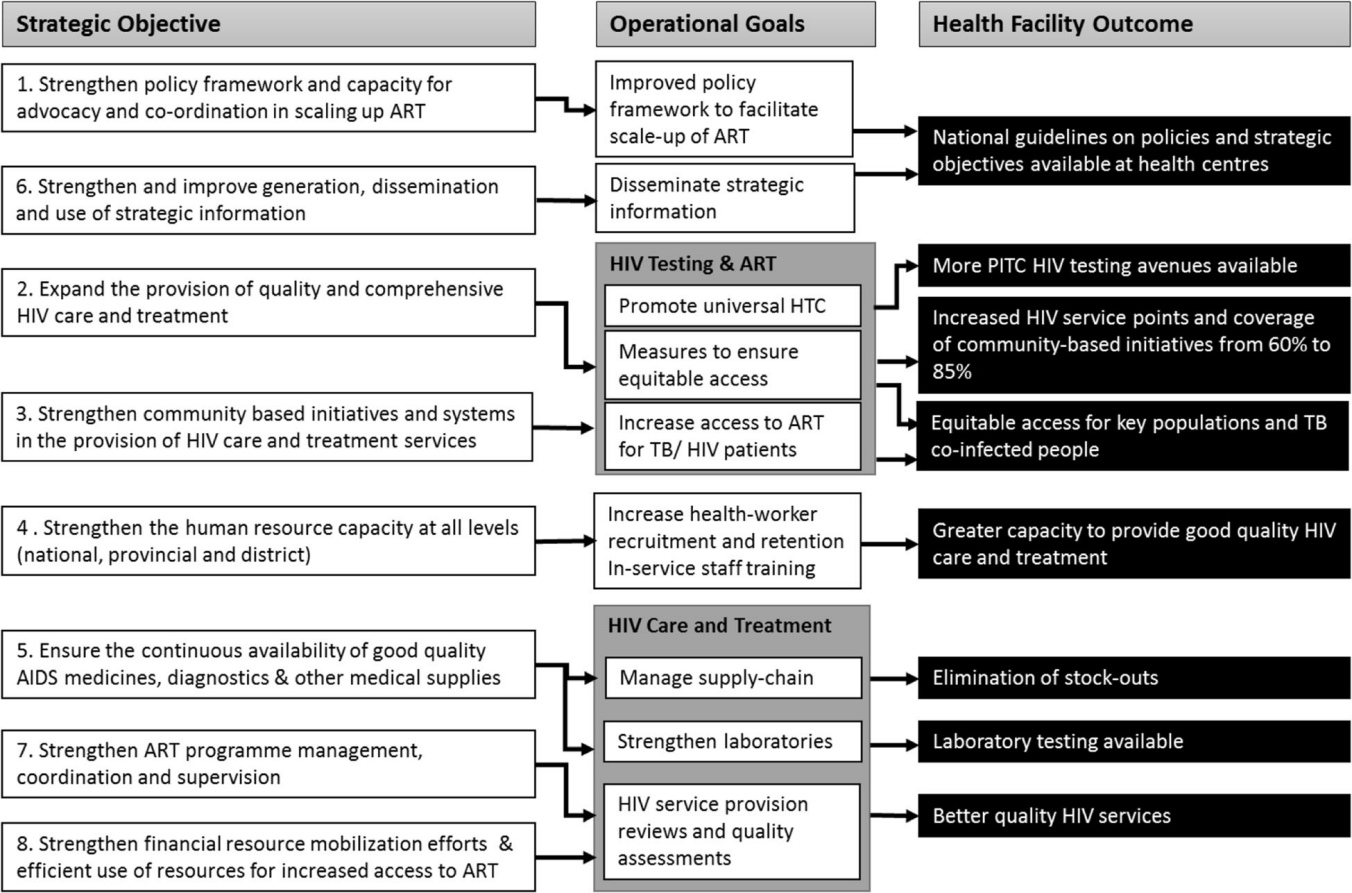
Zimbabwe strategic and operational plan for universal access to HIV services 2013–2017 [29]

An initial review of national policies, programme guidelines and strategic documentation published between 2003 and 2013 was conducted through a library search and online collation of policy documents pertaining to the provision of HTC, PMTCT and ART. The extraction of policy data was guided by a conceptual framework of indicators influencing the uptake of, and access to, HIV services developed by the Analysing Longitudinal Population-based data on HIV in Africa (ALPHA) Network [14, 30] and a set of eight objectives and operational goals identified as areas of strategic importance in HIV service delivery by the Zimbabwe Ministry of Health and Child Care (Fig. 1) [29]. In 2015, this review was extended to include documents published in 2014 and 2015. Ministerial departments were approached where further characterisation of policies was needed.

Policy data, including the policy/strategy prompt/question, the documented response, the policy document year of publication and source, the nature and dates of any changes in policy over the previous 10 years, and the presence of any policy conflicts, were abstracted into an Excel spreadsheet and analysed manually for the presence or absence of an explicit national policy or implementation strategy [14, 30]. Data were then coded to capture policy changes for pertinent indicators. Policies were grouped by service unit (HTC, PMTCT and ART provision) and by implementation characteristics, namely (1) service access and coverage, (2) quality of care provided, (3) co-ordination of care and patient tracking, (4) support provided to PLHIV, and (5) medical management of those accessing treatment. These policies were then compared to WHO recommendations.

### Policy implementation at health facilities

To assess levels, patterns and changes in local implementation of national policies, data were taken from two rounds of a longitudinal health facility survey. This was conducted by the Manicaland Centre for Public Health Research [31] in two predominantly rural administrative districts in the Manicaland Province (Makoni and Mutasa District) [31]. The survey included a sub-set of 36 health facilities selected from a baseline census of all HIV/AIDS service providers in the study districts. All district and other major hospitals (*n* = 5) were included in the sample, together with random samples of one-third of other types of health facility (large health centres (LHC) (*n* = 10), small health centres and clinics (SHCC) (*n* = 21)). Each round of the survey included a face-to-face interview with the manager or a senior member of staff at each facility and assessed the availability of human resources at the facility, the fees for and detailed components of HTC, PMTCT and ART services provided, the number of patients receiving care at the institution, and the bottlenecks perceived to be hindering service delivery.

Data on HIV services from the health facility surveys were collated and descriptive statistics calculated to estimate the proportion of facilities providing each HIV service component. For continuous variables, the means, medians and interquartile ranges were calculated. In the survey, questions pertaining to the delivery of ART were limited to those facilities that were ART initiation sites at the time of data collection. For indicators with missing data, the denominator used included only those facilities with data. Coverage was estimated for all surveyed facilities in the first instance and later stratified by the type of health facility (hospitals, LHCs and SHCCs). Comparisons were then made across the two survey rounds by calculating percentage point changes in coverage.

The availability of human resources in 2013 and 2015 was compared by assessing the number of healthcare workers in full-time and part-time employment at each facility. Healthcare workers were divided into five groups, namely (1) medical doctors, (2) registered nurses and midwives, (3) clinical officers, (4) nursing assistants/nursing aides and (5) laboratory staff. Uptake of HIV services was estimated by assessing four service categories, including (1) the number of HIV tests conducted over the previous 3 months, (2) the number of PMTCT visits recorded over the prior 3 months, (3) the number of visits made to the health facility by adult clients for pre-ART monitoring and assessment over the previous 3 months, and (4) the number of adult clients receiving first or second line ART at the health facility. In addition, district-level estimates of the numbers of healthcare workers and the uptake of HIV services were generated by weighting the data for LHCs and SHCCs to allow for the initial sampling of one-third of these facilities and to account for facilities with missing data.

To investigate differences in continuous outcome variables, the paired samples *t* test was used, assessing the difference in means between the two survey time points. Two-sided *p* values are reported. Differences in proportions were assessed using the McNemar test for correlated proportions to assess changes in the discordant pairs. Missing data was assumed to be missing completely at random. A complete case analysis was used in significance testing and thus only facilities with complete data for both study rounds were included. ART delivery proportions were limited to those facilities that provided ART at the time of the survey. However, in estimating the mean (adjusted and non-adjusted) number of clients receiving first- and second-line ART, all facilities were included. All monetary values are expressed in United States dollars.

## Results

### Policy reviews

A total of 10 national policy documents were reviewed in 2013. These comprised a training manual for an HTC course [32], the national HTC guidelines for 2005 [33], technical documentation on PMTCT provision [34], two community and home-based care guidelines [35, 36], the patient booklet/care register [37], national guidelines for tuberculosis (TB) treatment [38], the national essential medicines registry [39], the chronic HIV care pre-ART register [40], and guidelines on the use of ART for the prevention and treatment of HIV in Zimbabwe [12]. In addition, seven individuals were approached for clarification. In the 2015 policy review, five new policy documents were evaluated, including new guidelines on the use of ART for the prevention and treatment of HIV [13], new national guidelines on HTC [41], a policy document on the integrated management of adolescent and adult illnesses [42], the Zimbabwe national HIV operational and service delivery manual [43], and guidelines for the decentralisation of services for opportunistic infections (OIs) and ART [44]. In addition, one individual was approached for further policy clarification.

New national guidelines on the use of ART in Zimbabwe were released in November 2013, in which two key changes in national HIV treatment policy were noted. These were the introduction of Option B+ for PMTCT and an increase in the CD4 count eligibility threshold for ART initiation for non-pregnant adults from ≤ 350 cells/mm^3^ in 2010 to ≤ 500 cells/mm^3^ in 2013, both in line with WHO recommendations [8, 12]. Prior to this, ART eligibility for pregnant women was limited to those in WHO stage III or IV of HIV disease or a CD4 cell count equal to or below 350 cells/mm^3^. Amongst those who were not eligible for ART for their own health, antiretrovirals for PMTCT were prescribed up to 7 days postpartum [12]. In addition, the preferred first-line ART regimen for adult patients which, prior to 2013, was comprised of tenofovir (TDF) + lamivudine (3TC) + neviripine (NVP), or zidovudine (AZT) + 3TC + NVP, was changed to TDF + 3TC + efavirenz (EFV)/NVP, in line with the 2013 WHO treatment guidelines [8].

Clinical monitoring is a key component of measuring an individual’s response to ART. In its 2013 guidelines, WHO recommended CD4 count testing at ART initiation and 6-monthly thereafter [8]. This was synonymous with the national policies in Zimbabwe in both 2013 and 2015, where 6-monthly CD4 testing was also recommended for those stable on ART. WHO also recommended HIV viral load (VL) testing at 6 months after initiating ART, and every 12 months thereafter [8]. Laboratory tests that were desirable, where feasible, were haemoglobin testing for those initiating AZT, serum creatinine testing for those initiating TDF and an alanine aminotransferase test for those receiving NVP to evaluate liver function [8], further highlighting the need to monitor renal function where TDF is used [8]. In Zimbabwe, a number of national policies and strategies on the clinical monitoring of PLHIV existed prior to 2013. Laboratory tests that were deemed preferable but not essential before commencing ART were CD4 cell count testing, a full blood count for those receiving AZT, alanine aminotransferase, creatinine test where TDF is used and chest x-rays to exclude TB infection [12]. These similarities indicate a high uptake of WHO recommendations into Zimbabwe national treatment policies. In 2013, the Zimbabwe national guidelines changed to include the use of GeneXpert for TB screening before ART initiation, syphilis serology testing as well as screening for hepatitis B and C [13].

Other changes to the Zimbabwe national guidelines between 2013 and 2015 were the use of targeted repeat testing for HIV-negative high-risk individuals and the use of prophylactic isoniazid preventative therapy (IPT) against TB [13]. In addition, monitoring of ART adherence was introduced into national guidance (including provision of psychosocial counselling, supply of adherence cards and the requirement for patients to bring medications to all visits). National recommendations for the provision of co-trimoxazole prophylaxis (CTX) remained unchanged between 2013 and 2015, with CTX suggested for all patients with WHO stages II, III and IV and those with CD4 counts of ≤ 350 cells/mm^3^ [13]. However, this slightly differed from WHO guidance that recommended CTX for any WHO stage and CD4 count ≤ 350 cells/mm^3^, or WHO stage III and IV irrespective of CD4 [8].

In a 2008 policy update, WHO had recommended task-shifting as a strategy to increase coverage of HIV care and treatment services [45]. This was updated in 2013 to allow trained and supervised lay providers – non-physician clinicians, midwives and nurses – to initiate, distribute and maintain individuals on ART [8]. In addition, trained and supervised community health workers could dispense ART between regular clinical visits [8]. Switching of treatment from first- to second-line ART would continue to be performed by medical doctors. An explicit policy of multi-tasking and task-shifting of HIV care was adopted in Zimbabwe in May 2010 [12] and further outlined in the Zimbabwe National Strategic Plan for 2011–2015 [46]. New strategies were adopted in July 2013 to implement these policy changes, including increasing health worker capacity to provide HIV care and treatment in the form of in-service staff training and improved staff retention (Fig. 1) [29]. Comprehensive in-service training activities to improve competence in ART delivery across a broad range of staff were introduced, and special emphasis was placed on training nurses for their expanded role in ART initiation [29]. In addition, the decentralisation of key HIV activities was supported through strategies to increase investments in health and community systems and to improve community-based access to services, and services for key populations were extended (Fig. 1) [29].

### Policy implementation at health facilities

The health facilities included in the survey were managed by the Zimbabwe national government (*n* = 10), faith-based or private institutions (*n* = 11), or the rural district council (*n* = 15). Between 2013 and 2015, there were substantial reductions in healthcare workers overall (Table 1); although there was a slight increase in the mean number of medical doctors, this was counter-balanced by significant reductions in the mean numbers of registered nurses (RN) and midwives (2013: 8.72; 2015: 6.47; *p* = 0.04) and nurse aides (2013: 4.56; 2015: 3.61; *p* = 0.04). For RNs and midwives, reductions were seen across all types of health facility, whilst, for nurse assistants and aides, reductions were found predominantly at smaller facilities (LHCs and SHCCs) (Table 1). For both districts combined, weighted numbers of staff within the five analysis categories were estimated to have fallen from 788 in 2013 to 570 in 2015. Reductions were found in the mean numbers of RNs and midwives (2013: 4.37; 2015: 3.23), nursing assistants (2013: 3.41; 2015: 2.29) and laboratory staff (2013: 0.09; 2015: 0.04). A slight increase in the mean number of medical doctors was observed, rising from 0.14 in 2013 to 0.16 in 2015 (Table 3).

**Table 1:** Changes in health care staff in 2013 and 2015 in a sample of 36 facilities

	2013				2015				
	*N*	Mean	Median	Interquartile range	*N*	Mean	Median	Interquartile range	*p* value
Medical Doctors	14				16				0.16
Hospitals		2.6	2	(2–3)		3	3	(2–3)	
Large Health Centres		0	0	(0–0)		0	0	(0–0)	
Small Health Centres/Clinics		0.05	0	(0–0)		0.05	0	(0–0)	
Registered Nurses and Midwives	314				233				0.04
Hospitals		51.4	47	(18–61)		38.2	35	(13–38)	
Large Health Centres		2.5	1	(1–3)		1.3	1	(1–2)	
Small Health Centres/Clinics		1.52	1	(1–2)		1.38	1	(1–2)	
Clinical Officers	1				1				N/A
Hospitals		0.2	0	(0–0)		0.2	0	(0–0)	
Large Health Centres		0	0	(0–0)		0	0	(0–0)	
Small Health Centres/Clinics		0	0	(0–0)		0	0	(0–0)	
Nursing Assistants/Nursing Aides	164				130				0.04
Hospitals		15.8	16	8		16.6	16	(9–19)	
Large Health Centres		3.8	3	(2–6)		1.7	1	(1–2)	
Small Health Centres/Clinics		2.24	2	(1–3)		1.43	1	(1–2)	
Laboratory Staff	5				4				0.57
Hospitals		0.6	1	(0–1)		0.8	1	(1–1)	
Large Health Centres		0.2	0	(0–0)		0	0	(0–0)	
Small Health Centres/Clinics		0	0	(0–0)		0	0	(0–0)	

#### HIV testing and counselling

Table 2 summarises the coverage of HTC policy indicators. The proportion of health facilities offering free HTC increased from 86% in 2013 to 100% in 2015 (*p* = 0.03). Coverage of provider-initiated counselling and testing (PITC) for clients accessing antenatal care, TB and family planning clinics remained consistently high (≥ 97%) in both surveys. The mean number of HIV tests conducted per month through PITC increased for antenatal care clients (2013: 49.8; 2015: 73.5) and clients in the out-patient departments (2013: 66.4; 2015: 210.1). Decreases were observed in PITC testing for TB clients (2013: 5.3; 2015: 3.26) and clients accessing the sexually transmitted infections or family planning clinics (2013: 17.5; 2015: 4.2).

**Table 2:** Coverage of Zimbabwe national policies on HIV testing and counselling (HTC), prevention of mother to child transmission (PMTCT) and antiretroviral therapy (ART) care in 2013 and 2015

Number of facilities surveyed	Total facilities			Hospitals		Large health centres		Small health centres/clinics	
	2013	2015		2013	2015	2013	2015	2013	2015
	36 (100)	36 (100)	*p* value	5 (100)	5 (100)	10 (100)	10 (100)	21 (100)	21 (100)
HTC									
Service Access and Coverage									
Free HTC services	31 (86)	36 (100)	0.03	5 (100)	5 (100)	8 (80)	10 (100)	18 (86)	21 (100)
PITC to ANC clients	34 (97)^a^	35 (100)^a^	N/A	5 (100)	5 (100)	10 (100)	10 (100)	19 (95)^a^	20 (100)^a^
PITC to TB clients	36 (100)	34 (100)^a^	N/A	5 (100)	5 (100)	10 (100)	9 (100)^a^	21 (100)	20 (100)^a^
PITC to STI and FP clients	35 (100)^a^	35 (100)^a^	N/A	5 (100)	5 (100)	10 (100)	10 (100)	20 (100)^a^	20 (100)^a^
Testing offered to sex workers	21 (60)^a^	21 (58)	0.74	5 (100)	5 (100)	7 (78)^a^	6 (60)	9 (43)	10 (48)
Occurrence of at least one test-kit stock-out	25 (69)	22 (61)	0.44	5 (100)	4 (80)	7 (70)	4 (40)	13 (62)	14 (67)
Quality of Care									
≥ 1 staff member received HTC training in past 2 years	22 (63)^a^	18 (50)	0.35	5 (100)	4 (100)	6 (60)	5 (50)	11 (55)^a^	9 (43)
Quality of care audit at least once a year	32 (91)^a^	32 (89)	0.71	5 (100)	5 (100)	8 (80)	10 (100)	19 (95)^a^	19 (81)
Co-ordination of care and patient tracking									
Repeat test after 3-month period	35 (97)	26 (72)	0.01	4 (80)	4 (80)	10 (100)	6 (60)	21 (100)	16 (76)
Support to PLHIV									
Pre-test counselling always provided	35 (97)	34 (94)	0.56	5 (100)	5 (100)	10 (100)	8 (80)	20 (95)	34 (94)
Post-test counselling always provided	35 (97)	35 (97)	N/A	5 (100)	5 (100)	10 (100)	9 (90)	20 (95)	21 (100)
PMTCT									
Service Access and Coverage									
Free PMTCT services	16 (44)	35 (97)	< 0.001	1 (20)	5 (100)	6 (60)	10 (100)	9 (44)	20 (95)
Medical Management									
Co-trimoxazole prophylaxis available and in stock	33 (92)	34 (97)^a^	0.32	5 (100)	5 (100)	7 (70)	10 (100)	21 (100)	19 (95)^a^
Option B+	27 (79)^a^	35 (100)^a^	0.01	5 (100)	5 (100)	5 (56)^a^	10 (100)	17 (85)^a^	20 (100)^a^
PMTCT regimen (TDF, 3TC, EFV)	6 (18)^a^	35 (100)^a^	< 0.001	2 (40)	5 (100)	1 (13)^a^	9 (100)^a^	3 (14)	21 (100)
ART and Retention in Care									
Service Access and Coverage									
Free ART services	29 (81)	36 (100)	0.01	3 (60)	5 (100)	8 (80)	10 (100)	18 (86)	21 (100)
ART initiation at facility	14 (39)	35 (100)^a^	< 0.001	5 (100)	5 (100)	4 (40)	10 (100)	5 (24)	20 (100)^a^
Quality of Care									
Quality of care audit at least once a year	36 (100)	34 (100)^a^	N/A	5 (100)	4 (100)^a^	10 (100)	10 (100)	21 (100)	20 (100)^a^
≥ 1 staff member received HIV treatment training in past 2 years	9 (25)	19 (54)^a^	0.01	4 (80)	4 (80)	2 (20)	3 (33)^a^	3 (14)	12 (57)
Co-ordination of care and patient tracking									
Drugs collectable by designee	14 (100)	32 (91)^a^	0.32	5 (100)	5 (100)	4 (100)	10 (100)	5 (100)	17 (85)^a^
Home visits conducted following poor adherence	4 (27)	12 (34)^a^	0.08	1 (20)	3 (60)	1 (25)	6 (60)	1 (20)	14 (70)^a^
Home visits conducted following missed visit	11 (85)^a^	10 (29)	0.005	3 (75)^a^	0 (0)	4 (100)	4 (40)	4 (80)	6 (30)^a^
Laboratory Testing									
Laboratory testing services offered	9 (64)^b^	9 (26)	0.32	4 (80)	4 (80)	4 (100)	2 (20)	1 (20)^b^	3 (15)
Liver function tests conducted at facility	1 (7)	1 (3)	N/A	1 (20)	1 (20)	0 (0)	0 (0)	0 (0)	0 (0)
Renal function tests conducted at facility	1 (7)	1 (3)	N/A	1 (20)	1 (20)	0 (0)	0 (0)	0 (0)	0 (0)
HB/full blood count conducted at facility	7 (50)	7 (20)	0.32	4 (80)	4 (80)	2 (50)	0 (0)	1 (20)	3 (15)
CD4 testing conducted at facility	6 (46)^a^	6 (17)	N/A	4 (80)	4 (80)	2 (50)	2 (20)	0 (0)^a^	0 (0)
Medical Management									
Nurse-led ART initiation	10 (71)	32 (100)^a^	0.08	3 (60)	2 (100)^b^	3 (75)	10 (100)	4 (80)	20 (100)
WHO-recommended first-line regimen for non-pregnant adults	9 (64)	33 (94)	0.1	4 (80)	5 (100)	2 (50)	9 (90)	3 (60)	19 (95)
6 monthly (min) CD4 on ART stable patients	14 (100)	35 (100)	N/A	5 (100)	5 (100)	4 (100)	10 (100)	5 (100)	20 (100)
Prophylactic IPT in stock	11 (79)	15 (43)	0.41	5 (100)	5 (100)	3 (75)	3 (30)	3 (60)	7 (35)
TB screening at every ART visit	12 (86)	35 (100)	0.16	3 (60)	5 (100)	4 (100)	10 (100)	5 (100)	20 (100)
At least two first-line regimen choices available at facility	12 (92)^a^	15 (43)	0.02	5 (100)	2 (40)	2 (67)^a^	5 (50)	5 (100)	8 (40)
Occurrence of at least one stock-out of first-line ART drugs	1 (7)	0 (0)^a^	N/A	0 (0)	0 (0)	0 (0)	0 (0)	1 (20)	0 (0)
Occurrence of at least one stock-out of OI drugs	4 (21)	2 (6)	0.65	0 (0)	1 (20)	2 (50)	0 (0)	1 (20)	1 (5)

*3TC* lamivudine, *ANC* antenatal care, *EFV* efavirenz, *FP* family planning, *HB* haemoglobin, *IPT* isoniazid preventative therapy, *OI* opportunistic infection, *PITC* provider-initiated testing and counselling, *PLHIV* people living with HIV, *STI* sexually transmitted infection, *TB* tuberculosis, *TDF* tenofovir

^a^Under 10% data missing

^b^Over 10% data missing

Targeted testing offered specifically to sex workers, as well as the provision of staff training on HTC, remained marginal. There was a reduction in the proportion of facilities offering repeated testing after the 3-month window period for those who had tested HIV negative from 97% in 2013 to 72% in 2014 (*p* = 0.01); this was particularly pronounced in LHCs and SHCCs (Table 2). Despite this, counselling provided prior to and after HIV testing remained high across both survey rounds. Stockouts in HIV test kits on at least one occasion over the prior year were reported by a large proportion of facilities, both in 2013 (69%) and in 2015 (61%). The mean duration of the longest stock-out decreased from 54.5 days in 2013, to 17.9 days in 2014 (*p* = 0.0005). This reduction was observed amongst all three types of health facility, with a decrease of 31.1 days in hospitals, 44.7 days in LHCs and 36.4 days in SHCCs.

**Fig. 2:**
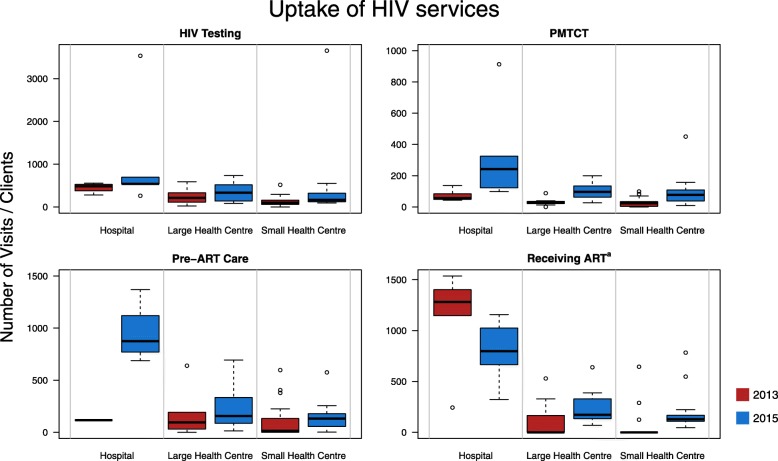
Uptake of HIV services across different types of health facilities in Eastern Zimbabwe. Sample sizes (*n*): **2013:** HIV Testing: Hospitals (*n* = 4), LHC (*n* = 10), SHC (*n* = 20); PMTCT: Hospitals (*n* = 5), LHC (*n* = 10), SHC (*n* = 21); Pre-ART: Hospitals (*n* = 1), LHC (*n* = 6), SHC (*n* = 16); ART: Hospitals (*n* = 5), LHC (*n* = 9), SHC (*n* = 19). **2015:** HIV Testing: Hospitals (*n* = 5), LHC (*n* = 10), SHC (*n* = 19); PMTCT: Hospitals (*n* = 5), LHC (*n* = 10), SHC (*n* = 19); Pre-ART: Hospitals (*n* = 5), LHC (*n* = 10), SHC (*n* = 20); ART: Hospitals (*n* = 5), LHC (*n* = 10), SHC (*n* = 20). Open circles in the box plots represent outliers. ^a^ For those receiving ART, all facilities were included in 2013, including those that were not yet delivering ART

Increases in numbers of HIV tests conducted were found across all types of health facilities (*p* = 0.035) (Fig. 2). The largest mean increase in HTC visits was seen in hospitals (2013: 451.3; 2015: 1113.4). Overall, the unadjusted mean number of HIV testing visits rose from 202.3 in 2013 to 488.9 in 2015. When extrapolated to district level, weight-adjusted numbers of HTC visits rose from 17,027 in 2013 to 38,735 in 2015, with higher volumes observed in Mutasa (2013: 9418; 2015: 23,615) than in Makoni (2013: 7609; 2015: 15,120). Weight-adjusted mean numbers of HTC visits per facility rose from 181.1 in 2013 to 421.0 in 2015.

#### Prevention of mother-to-child transmission (PMTCT) of HIV

In the first round of the survey, coverage of free PMTCT services was low (44% of health facilities). The median fee for PMTCT at the 20 facilities that charged for the service was US$ 10 (range US$ 1–30). By 2015, 97% of facilities offered free PMTCT (the one facility that required payment charged US$ 11). Progress in the provision of free PMTCT were found across all types of health facilities (Table 2). The availability of prophylactic CTX remained high between the two survey rounds, with marginal improvements observed within LHCs. The provision of Option B+ and the WHO recommended PMTCT regimen (TDF + 3TC + EFV) increased significantly over time (Table 2). Amongst facilities offering PMTCT services, 79% offered Option B+ in 2013, compared to 100% in 2015 (*p* = 0.01). Progress in increasing service coverage was particularly evident at LHCs where coverage of Option B+ increased from 56% in 2013 to 100% in 2015. Use of the recommended treatment regimen increased (2013: 18% vs. 2015: 100%) in line with national treatment policies (Table 2).

Unadjusted estimates of the number of PMTCT visits recorded in the 3 months prior to each survey showed a significant increase between 2013 and 2015 (2013: 34.7; 2015: 131.4; *p* < 0.001), with the largest mean increase observed amongst hospitals (2013: 73.8; 2015: 340). District estimates for the numbers of PMTCT visits rose from 2715 in 2013 to 9998 in 2015, whilst means rose from 27.7 in 2013 to 108.7 in 2015 (Table 3).

**Table 3:** District estimates of HIV service uptake and human resources

	Total facilities				Mutasa^a^				Makoni^a^			
	2013		2015		2013		2015		2013		2015	
	*N*	Mean	*N*	Mean	*N*	Mean	*N*	Mean	*N*	Mean	*N*	Mean
Uptake of HIV services												
HIV Testing and Counselling	17,027	181.1	38,735	421	9418	188.4	23,615	524.8	7609	172.9	15,120	321.7
PMTCT	2715	27.7	9998	108.7	1113	21.8	3299	73.3	1899	40.4	6699	142.5
Pre-ART care	9705	144.9	18,132	190.9	4479	93.3	7432	154.8	5226	275.1	12,422	264.3
ART	6310	123.7	9789	191.9	6310	123.7	9789	191.9	5911	125.8	12,438	264.6
Human resources												
Medical Doctors	14	0.14	16	0.16	8	0.16	9	0.18	6	0.13	7	0.15
Registered Nurses and Midwives	428	4.37	317	3.23	213	4.18	137	2.69	215	4.57	180	3.83
Clinical Officers	1	0.01	1	0.01	1	0.02	1	0.02	0	0	0	0
Nursing assistants and aides	334	3.41	224	2.29	189	3.71	137	2.69	145	3.09	87	1.85
Laboratory staff	9	0.09	4	0.04	6	0.12	3	0.06	3	0.06	1	0.02

^a^District population estimates from 2012 national census: Mutasa: 168,747, Makoni: 272,340 [47]

#### Access to antiretroviral therapy, patient retention and monitoring

The decentralisation of HIV treatment from hospitals to smaller clinics has led to a high proportion of facilities offering ART initiation and follow-up in 2015, increasing from 39% in 2013 to 100% in 2015 (Table 2). ART was provided freely in 2013, with the limitation, however, that administration costs could be requested up to a maximum of US$ 5. In 2015, national policies explicitly stipulated that HIV/AIDS treatment was free. This policy alteration was widely implemented amongst surveyed health facilities, with improvements seen between 2013 and 2015, wherein the provision of free ART rose from 81% to 100% (*p* = 0.01). ART service coverage increased, despite only a modest rise in the proportion of health facilities reporting that at least one staff member had received training in HIV treatment in the previous 2 years (from 25% in 2013 to 54% in 2015; *p* = 0.01). All facilities surveyed in 2013 reported annual audits of their HIV treatment programme despite there being no explicit national requirement to do so; however, these were stated in the national strategic objectives (Fig. 1), where improvements in the quality of care and treatment received by PLHIV is stipulated. Certification processes were outlined in the 2015 policy review, which would be undertaken by district clinical/ART mentorship teams with quality audits being done on a quarterly basis.

Amongst health facilities offering ART initiation, coverage of laboratory testing facilities to enable medical management of PLHIV was low in both rounds, despite national policy recommendations for tests to be conducted prior to ART initiation and the emphasis placed on the diagnostic capabilities in the national strategic and operational plan [25] (Fig. 1). The availability of CD4 count testing was provided by the same six health facilities in both rounds, even with the increase in ART initiation sites. Despite this limited coverage, the proportion of health facilities that reported offering 6-monthly CD4 counts for PLHIV stable on ART remained consistently high (100%). The provision of liver and renal function tests remained confined to hospitals only. Coverage of haemoglobin and full blood count testing was also partial, with limited availability at LHCs and SHCCs (Table 2). The continuous availability and supply of quality HIV treatment diagnostics and medicines was further assessed through the occurrence of stock-outs of first-line ART drugs and prophylactic drugs for OIs, specifically CTX, fluconazole and IPT. This was evaluated over the 12-month period prior to data collection. The proportion of facilities that reported at least one stock-out of first-line ART drugs, such that they could not provide treatment for patients on ART, remained consistently low. Only one facility reported at least one stock-out of first-line ART drugs in 2013, with the longest stock-out lasting 30 days. No stock-outs of ART drugs were reported in 2015. Those reporting at least one stock-out of drugs for OIs over the previous 12 months, such that they could not provide medication, decreased from 21% in 2013 to 6% in 2015 (Table 2). Despite national policies on the use of IPT for the prevention of TB, coverage decreased from 79% in 2013 to 43% in 2015 (*p* = 0.41), falling predominantly within LHCs and SHCCs.

Amongst strategies to aid the co-ordination of care and patient tracking activities, national guidelines and policy documents suggested that ART prescriptions could be collected by a designated person such as a supporter or treatment buddy or a family member. Coverage of this implementation strategy was high both in 2013 and 2015, at 100% and 91% of facilities, respectively (*p* = 0.32). Other strategies aimed at reducing patient attrition include the use of home visits following poor adherence and tracing of those defaulting on treatment (less than 90 days). No national policies were stipulated with regards to home visits following poor adherence, however, an increasing proportion of facilities used this strategy, rising from 27% in 2013 to 34% in 2015 (*p* = 0.08). The use of village health workers through community outreach initiatives was encouraged in 2013 to trace defaulters and home visits were stipulated in 2015; however, coverage fell over the two survey periods (2013: 85%; 2015: 29%; *p* = 0.005), especially amongst hospitals (Table 2).

The explicit policies and strategies for multi-tasking and task-shifting for ART initiation, as previously mentioned, translated into an increase in coverage of nurse-led ART initiation amongst the surveyed health facilities from 71% in 2013 to 100% in 2015 (*p* = 0.08). The proportion of facilities offering the WHO recommended first-line ART regimen for non-pregnant adults also increased from 64% in 2013 to 94% in 2015 (*p* = 0.1); however, availability of at least two first-line ART choices decreased from 92% in 2013 to 43% in 2015 (*p* = 0.02), suggesting that difficulties may be encountered should stock-outs occur. Routine screening for TB amongst PLHIV is explicitly stated in national policy documents and coverage remained high within health facilities (Table 2), despite a growth in the number of PLHIV enrolled in care between 2013 and 2015.

Unweighted estimates of the mean number of pre-ART visits across all surveyed health facilities increased from 144 in 2013 to 281 in 2015 (*p* = 0.68), with the largest increases observed amongst hospitals (Fig. 2). Weight-adjusted estimates of district level changes in the number of pre-ART visits showed an overall increase from 9705 in 2013 to 18,132 in 2015. Increases were higher in Makoni district (2013: 5226; 2015: 12422) compared to Mutasa (2013: 4479; 2015: 7432). The adjusted mean number of pre-ART visits per facility increased from 144.9 in 2013 to 190.9 in 2015.

The average number of clients receiving first- or second-line ART increased overall (2013: 244.15; 2015: 286.9; *p* = 0.2). When stratified by health facility type, the largest increase was observed within SHCCs (2013: 55.6; 2015: 183.2), followed by LHCs (2013: 127.4; 2015 241.4). The mean number of ART clients fell in hospitals from 1121.8 in 2013 to 793.2 in 2015. The total estimated number of ART clients in the two districts rose from 12,221 in 2013 to 22,197 in 2015; rising more prominently in Makoni district (increase of 6527) than in Mutasa district (increase of 3479). District estimates for the mean numbers of clients per facility increased from 137.3 in 2013 to 233.7 in 2015 (Table 3) [47].

## Discussion

In a review of national HIV care and treatment guidelines in Zimbabwe, we found high uptake of WHO recommendations adopted into national policy. New national HIV treatment guidelines were produced in Zimbabwe in November 2013 following the release of new WHO recommendations in June 2013. Changes adopted included an increase in the CD4 count threshold for ART eligibility, the introduction of Option B+, and a change in the recommended first-line ART regimen. Between 2013 and 2015, we found evidence of increases in implementation of pre-existing and new national policies at facility level over time. Notable improvements included increases in the proportions of health centres offering free HTC, PMTCT and ART services, increased coverage of Option B+, and use of the latest WHO recommended regimens for pregnant women and non-pregnant adults.

For some indicators, service provision went beyond the standards outlined in explicit policies (e.g. annual quality of care audits and, at some facilities, home visits in cases of poor ART adherence), whilst, for a few others, implementation remained low or decreased (e.g. gaps in the availability of prophylactic IPT and in coverage of pre-ART initiation laboratory tests), despite the emphasis on strengthening local diagnostic capabilities within the national strategic objectives [29]. Our results also highlighted the existence of implementation gaps in the availability and stocking of at least two first-line ART regimen choices. Whilst the WHO-recommended first-line ART regimen of TDF + 3TC + EFV/NVP has been adopted into Zimbabwe national guidelines, the limited availability of different regimen choices may provide challenges in the provision of ART should stock-outs occur or alternate regimens be required. Similar findings on stock-outs within public and private sector ART sites from all administrative districts in Zimbabwe have been reported, where only 18.9% of sites included reported no ART stock-outs for adult treatment regimens [48]. Similarly, the gaps observed in the provision of prophylactic medicines such as CTX and IPT are of concern as adequate supply chain management has been identified as a bottleneck in the provision of ART [24].

We found that the general improvements in service provision at the facility level translated into large increases in the numbers of visits made by clients for HIV testing and treatment services. This picture is supported by data from a longitudinal general population open cohort survey in the same study districts in Manicaland province, showing steady improvements in all stages of the treatment cascade between 2008 and 2013 [49]. Strategies that may have contributed to increases in facility implementation include decentralisation of services to more rural facilities, nurse-led ART initiation, and increased training of health workers in the provision of HIV care and treatment. These strategies appear to have been implemented successfully in east Zimbabwe, despite substantial reductions in numbers of health workers. One factor that may have contributed to this is the results-based funding (RBF) system – a health system financing strategy based on performance and outputs at facility level – that was introduced in Zimbabwe in 2011. The RBF system is aimed particularly at improving service delivery at rural outposts [50]. Although not initially aimed specifically at improving HIV services, the availability of additional funds and overall strengthening of the health system may have played a role in enhancing these services [51].

Whilst it is known that multiple sources exist, including RBF and the country’s own internally funded National AIDS Trust Fund [52], and that resources generally have been severely constrained by the continued weakness of the national economy, this study did not investigate funding streams for HIV and other health programmes in Zimbabwe. However, detailed research on specific uses of different funding streams at the facility level could be beneficial to identify means to address the gaps in policy implementation highlighted in this study. Particular attention could be paid to investigating the priorities and activities set by non-governmental organisations and external donors, and their influence on patterns of implementation. In a 2017 analysis assessing policy implementation across two Health and Surveillance Sites in Uganda [48], key informants revealed the need for commitments from external partners to provide the resources required to support implementation; such issues may be a concern in Zimbabwe as well, thus warranting further investigation.

Progress with implementation of national HIV policies has also been evaluated in a cross-sectional study of six health facilities in Karonga, Malawi, in December 2013 [53, 54]. Free HTC, PMTCT and ART services were provided at all facilities and Option B+ was available in 90% of facilities, suggesting slightly earlier implementation of these services in Malawi compared to east Zimbabwe. The later implementation of updated HIV services found in east Zimbabwe compared to some of the sites in other countries may be due, in part, to the economic difficulties that the country continues to face following the economic collapse in 2009 [55]. For example, these economic difficulties may have contributed to the falling numbers of health workers we observed in this study. However, in general, our findings for east Zimbabwe fit a wider pattern of substantial overall progress in adoption and implementation of WHO recommendations on HIV testing and treatment services, albeit with some important gaps remaining (e.g. services for high-risk groups) [56].

Prior to the scale-up of ART services from the mid-2000s, Zimbabwe experienced an earlier and larger reduction in HIV prevalence [25, 26] compared to neighbouring countries such as Botswana and South Africa. This relative success was achieved during a period of limited external donor support and has been attributed primarily to spontaneous local responses to high AIDS mortality backed up by effective national HIV prevention programmes [25]. In contrast, the scale-up of ART services in Zimbabwe has received greater donor support, with donor funds accounting for 75–100% of the total HIV/AIDS budget in 2013 [14], much higher than in South Africa, for example, where the equivalent figure was 0–24% [14]. External partners may be more willing to take up new strategies to expand the provision of HIV care and treatment services than national governments, which may be more parsimonious. Possibly as a consequence, as we document here, national ART policies in Zimbabwe have largely followed, and sometimes have surpassed, WHO recommendations. Examples of the latter include the early adoption of home-based care policies [57] and introduction of services for anonymous HTC, training for healthcare staff and patient tracking following ART initiation [14].

In December 2016 – subsequent to the period covered by the current study – the Government of Zimbabwe adopted the new WHO guidance recommending ART initiation for all HIV-positive individuals regardless of CD4 count or clinical stage and the UNAIDS 90–90–90 targets. To achieve these new targets and to address pre-existing gaps in policy implementation, more innovative strategies for service implementation will be needed. Current initiatives in Manicaland and in other parts of Zimbabwe include revival of health centre committees to increase community involvement, mobile HIV testing, and index case-based HIV testing. Other strategies that could be extended or are being piloted and could be effective include HIV self-testing [58, 59] and home visits for people with poor ART adherence. Policies pertaining to retention of patients in care and innovative strategies to improve patient monitoring will be important for achieving high levels of virological suppression – the last of UNAIDS 90–90–90 treatment goals. One key component that will be needed is VL monitoring to allow for timely detection of treatment failure. Despite the recent introduction of VL monitoring into national policy in a number of countries in sub-Saharan Africa, implementation of VL monitoring remains limited and is targeted largely towards high risk individuals [60, 61]. Previous studies have found higher rates of switching to second-line ART amongst individuals in Uganda and in Zimbabwe that received both clinical and laboratory monitoring, as opposed to laboratory monitoring alone [62]. This highlights the importance of national policies aimed at improving patient monitoring coupled with providing innovative methods of service delivery both in and out of the health facilities to further decentralise services, particularly laboratory services, to limit patient attrition.

Limitations of this study include missing data for some indicators of policy implementation (as indicated in Table 2), which may distort the comparison between time periods as a complete case analysis was used to generate statistical estimates. Furthermore, the study was conducted in two predominantly rural districts of Manicaland, which is currently the province with the lowest HIV prevalence in Zimbabwe [63].

## Conclusion

Through the progressive uptake of WHO recommendations for ART initiation into national HIV care and treatment policies, coverage of PMTCT Option B+ and ART use has increased in Zimbabwe between 2013 and 2015. This may be due not only to extensions in ART eligibility but also to implementation of key strategies, including the decentralisation of service provision from hospitals to clinics located closer and within communities and task- shifting of HIV care to lower-level cadres of healthcare workers within the health system. Coverage of PITC initiatives has been consistently high, although specific provision for high-risk groups, such as men who have sex with men, remains limited. Whilst many facilities continued to provide mobile testing and outreach services, laboratory monitoring remained confined to larger hospitals. As the population of individuals requiring HIV services is projected to grow, further innovative service delivery strategies may be needed to improve linkage to care and to reduce losses to clinical follow-up. Community-based interventions, such as self-testing and home-based ART initiation, and clinical monitoring may be beneficial in achieving 90–90–90 treatment targets.

## Abbreviations

3TC: lamivudine
ART: antiretroviral therapy
AZT: zidovudine
CTX: co-trimoxazole
EFV: efavirenz
HTC: HIV testing and counselling
IPT: isoniazid preventative therapy
LHC: large health centre
NVP: neviripine
OI: opportunistic infection
PITC: provider-initiated testing and counselling
PLHIV: people living with HIV/AIDS
PMTCT: prevention of mother-to-child transmission of HIV
RBF: results-based funding
RN: Registered Nurse
SHCC: small health centres and clinics
TB: tuberculosis
TDF: tenofovir
UNAIDS: Joint United Nations Programme on HIV/AIDS
VL: viral load
